# PTEN: A Thrifty Gene That Causes Disease in Times of Plenty?

**DOI:** 10.3389/fnut.2020.00081

**Published:** 2020-06-05

**Authors:** Ajit Venniyoor

**Affiliations:** Department of Medical Oncology, National Oncology Centre, The Royal Hospital, Muscat, Oman

**Keywords:** PTEN (phosphatase and tensin homolog deleted on chromosome 10), thrifty gene hypothesis, insulin resistance, carcinogenesis, polycystic ovarian disease (PCOD), diabetes mellitus, NAFLD

## Abstract

The modern obesity epidemic with associated disorders of metabolism and cancer has been attributed to the presence of “thrifty genes”. In the distant past, these genes helped the organism to improve energy efficiency and store excess energy safely as fat to survive periods of famine, but in the present day obesogenic environment, have turned detrimental. I propose PTEN as the likely gene as it has functions that span metabolism, cancer and reproduction, all of which are deranged in obesity and insulin resistance. The activity of PTEN can be calibrated *in utero* by availability of nutrients by the methylation arm of the epigenetic pathway. Deficiency of protein and choline has been shown to upregulate DNA methyltransferases (DNMT), especially 1 and 3a; these can then methylate promoter region of PTEN and suppress its expression. Thus, the gene is tuned like a metabolic rheostat proportional to the availability of specific nutrients, and the resultant “dose” of the protein, which sits astride and negatively regulates the insulin-PI3K/AKT/mTOR pathway, decides energy usage and proliferation. This “fixes” the metabolic capacity of the organism periconceptionally to a specific postnatal level of nutrition, but when faced with a discordant environment, leads to obesity related diseases.

## Introduction

In the early sixties, Neel (1) suggested that evolution has selected some populations for “thrifty genes” to survive cycles of famines but these genes are now proving detrimental in an age of overabundance, leading to modern diseases such as obesity, metabolic syndrome, and cancer. Many genes have been proposed as the putative thrifty genes [see review by Prentice et al. (2)] but the evidence for a specific gene alteration generating a thrifty genotype is inconclusive (3). Hales and Barker (4) suggested a developmental alternative (30 phenotype)—that the fetus, when exposed to poor nutrition undergoes programming *in utero* to survive anticipated nutrition constraints in the post natal nutrition-poor environment also, but develops disease if the post natal environment is nutrition-rich. This has been backed with epidemiological data (such as birth records in England) and is the basis for the Developmental Origins of Health and Disease (DOHaD) concept (5–7). The role of epigenetics in programming the genome has gained prominence more recently and the argument has been made for a “thrifty epigenotype” (8).

The criteria for a potential thrifty gene has been laid out (2) and a crucial property is energy efficiency—to reduce energy expenditure and store excess energy in anticipation of periods of famine.

## The Obesity Epidemic

The classical hypothesis that the basis of obesity is energy imbalance (calorie in, calorie out) does not explain various features of the obesity epidemic [for excellent discussions, read (9, 10)]. The “carbohydrate-insulin model” of obesity suggests that the primary defect lies in fat storage; excess fat storage causes obesity first which is followed by increased intake (hunger) and reduced activity. Excess fat storage is driven by hyperinsulinemia [which in turn is driven by high glycemic index foods and food additives in processed foods (11)]. Insulin is essentially an anabolic hormone whose metabolic function is to store absorbed glucose as glycogen and fat. Food with high glycemic index (sugary and processed) that is widely available as part of the Western diet causes a spike in insulin which deposits glucose as fat (causing obesity with hypoglycemia and increasing hunger). Insulin also stimulates *de novo* lipogenesis (DNL), which is a feature of fatty liver; the latter can also be produced by fructose (from high sugar intake) metabolism in liver (12). It follows that any alteration that makes the insulin-glucose pathway more active will result in efficient fat storage, even at low or normal insulin levels. PTEN is a protein that sits astride the metabolic [phosphoinositide 3-kinase (PI3K)-AKT-mammalian target of rapamycin (mTOR) (PI3K-AKT-mTOR)] pathway of insulin and suppresses it. Reduced activity of PTEN will smoothen the passage of signals along this pathway, causing “relative hyperinsulinemia.”

## Hypothesis

It is proposed that PTEN is the primary thrifty gene. Epigenetic changes *in utero* due to deficiency of specific nutrients is the basis for fetal programming for a nutrient-poor postnatal environment. The modern epidemic of metabolic diseases and obesity related cancers (ORCs) results when a PTEN deficient organism, programmed for a nutrient-poor environment, with limited metabolic capacity, is exposed to nutrient-rich surroundings.

## The Gene

PTEN (phosphatase and tensin homolog deleted on chromosome 10) (13, 14) was initially recognized as a tumor suppressor gene (TSG); it acts by suppressing the PI3K-AKT-mTOR proliferative pathway through its lipid phosphatase activity, therefore inhibiting cell proliferation. It was later found to be involved in a wide spectra of other cellular processes including energy metabolism (as it sits downstream of the insulin pathway and negatively regulates it, and also alters mitochondrial functioning), survival, proliferation, and cellular architecture. PTEN has certain interesting properties such as (15, 16):

- It is dominant negative: the inactive PTEN mutants hetero-dimerise with wild-type PTEN and reduce its phosphatase activity.
- It is haploinsufficient: One copy of the gene is insufficient for proper function. Loss-of-function mutation results in a situation where the amount of protein product created from the remaining wild-type allele is not sufficient for normal cellular function.
- It is quasi-insufficient (17): it cannot sustain adequate biological functions upon a subtle reduction in the protein levels. Marginal reductions in PTEN expression (“dose”) (18) along the mitogenic pathway gives rise to a variety of cancers; this hypothesis implies that similar dose modifications can exist along the metabolic pathway with lower doses making the pathway more efficient (resulting in storage of more fat at equivalent levels of insulin).

The expression and function of PTEN can be modified in a variety of ways, ranging from gene mutations, epigenetic regulation by promoter methylation, post transcriptional modifications and by microRNAs (19).

It is proposed that deficiency of specific nutrients (proteins especially methionine, and choline) leads to upregulation of DNA methyltransferases (DNMT3a, possibly DNMT1) by hypomethylation; this leads to promoter methylation of PTEN and varying degrees of suppression (proportional to the availability of the specified nutrients), resulting in fetal programming. Thus, nutritional supply decides the degree of PTEN expression (“dose”) and the thrifty phenotype, but when faced with post natal nutrition-rich environment, can lead to modern diseases such as metabolic syndrome and cancer.

There are certain questions that need to be answered in support of this hypothesis.

1. Is it the calorie restriction *per se* or lack of specific nutrients that lead to epigenetic changes? Interest in the field of nutritional epigenetics is expanding and there are multiple epidemiological studies and experimental animal models addressing the fetal and transgenerational outcomes of maternal dietary manipulation. Epigenetic changes, especially DNA methylation, in response to changes in maternal nutrition has been well documented (20). Calorie restriction *per se* has been noted to lead to changes in gene expression (notable research being carried out in the hope of extending life span) but current interest is in teasing out the specific components of diet that contribute to altered gene expression. The current concept of “protein leverage (21) suggests that most important part of diet is protein, especially the essential amino acid methionine, and the body can sense and respond to protein and methionine deficiency (via FGF21) (22). However, methionine levels are tightly controlled in the fetus (23) as befitting its importance, and other one-carbon donors such as choline and folates can step in as needed to regulate DNA methylation (24). It is thus suggested that choline is also an important factor in fetal (mal)nutrition that decides the degree of methylation of PTEN and thus the level of its protein expression. Consistent with this hypothesis, maternal low protein diet produced low Pten expressing mouse mammary Tumors in the offspring (25). The effect of low protein or low choline on normal tissue has not been reported to my knowledge.
2. Which are the DNA methyltransferases (DNMT) sensitive to nutritional changes? DNMTs are responsible for methylation of promoter regions and usually repress gene expression; the main ones are DNMT1, 3a and 3b. DNMT3a is consistently upregulated in calorie (26), protein (27–29), methionine (30), and choline deficient states (31). Gong et al. reported that both the expression levels of DNMT1 and DNMT3a were significantly increased when pregnant rats were fed a low-protein diet (27). Choline deficient diet in mother resulted in upregulation of both Dnmt1 and 3a in offspring in another study (31). There are multiple studies using combination deficiencies such as the choline deficient, amino acid defined (CDAA) diet (32), methionine-choline deficient (MCD) (33) diet and folate-methyl deficient (FMD) (34) models where the results are generally consistent with upregulation of one or both DNMTs.
3. Does alteration in maternal diet lead to changes in PTEN expression in the offspring? As mentioned above, low protein diet in dams can result in downregulation of PTEN in the offspring mammary Tumors (25). Food restriction *per se* has been shown to downregulate Pten in fetal liver (35). However, in another study of calorie restriction, Pten expression was not changed in skeletal muscles (though Akt was activated) (26). Methionine restriction (MR) has also been shown to keep PTEN inactive by reducing the amount of GSH need to keep it in an active, reduced state (30). Choline deficiency downregulated the expression of Pten in the liver of mice fed choline deficient, amino acid defined (CDAA) diet (32). (Note that the latter studies were in adult mice; the effect of choline deficient maternal diet on offspring has not been studied to my knowledge). This is consistent with the fact that choline deficient diet leads to non-alcoholic fatty liver disease (NAFLD); a condition known to be associated with PTEN downregulation (36). However, the classical MCD (methionine-choline deficient) diet model of NASH is associated with PTEN upregulation in murine liver (37) [while a high fat diet (HFD) model of NASH results in downregulation of PTEN in liver (38)]. Interestingly, PTEN deficiency in adipose tissue protects against NAFLD (39), suggesting that the resultant more efficient insulin action could lead to preferential fat storage in adipose tissue, sparing ectopic fat deposition in liver. The expression and function of PTEN appears to be thus tissue and context dependent.
4. Has calorie/nutrient restriction been shown to result in downregulation of PTEN? There are large data sets from the survivors of famines [such as Dutch Winter Hunger (40), Chinese (41, 42) or Bangladeshi (43) famines] but the published data sets do not include status of PTEN. There is, however, evidence from cell lines and animal models.
  - Fasting caused (transient) inhibition of Pten in intestinal stem cells (44).
  - Calorie restriction reduced expression of Pten in silkworm larvae (45).
  - Maternal undernutrition reduced the expression of Pten in the liver of sheep offspring singletons (46).
  - Offspring of mice with gestational diabetes mellitus have raised Pten expression; calorie restriction downregulated this (47).
  - Pten was down regulated in fetal liver following a choline deficient, aminoacid defined (CDAA) diet in C57BL/6 mice (32).
  - Maternal protein deprivation resulted in upregulation of PI3K and GLUT4 in muscles of the offspring of rats (consistent with Pten deficiency, though this was not specifically reported) and increased insulin sensitivity into adulthood (48).
5. Has altered PTEN expression been demonstrated in normal human tissue? Defining the “normal dose” of the protein is impractical as it varies across tissues; studies usually compare the expression of the PTEN protein to that of a protein from another gene (by immunohistochemistry). Not surprisingly, most reported studies are from cancer patients, where expression of PTEN in Tumor tissue has been compared to adjacent “normal tissue”(which may not necessarily be normal) (49). There are scattered studies reporting loss in adjacent normal tissue in cancers of breast (50), endometrium (51), gallbladder (52), colon (53), esophagus (54), thyroid (55), and kidney (56).

Interestingly, these are also obesity related cancers (ORCs), a point to which I will return to later. It is, of course, not known whether these individuals with loss in normal tissue were nutritionally deprived *in utero*.

Information about methylated PTEN in normal tissue is even scarcer (57–61). There are several caveats about promoter methylation and resultant repression of the gene that is worth keeping in mind (62, 63).

- Epigenetic change is not an “all or none” phenomenon—a promoter can be partially methylated in precursor lesions and then progress to full methylation and complete gene silencing. As noted earlier (18), subtle suppression of PTEN protein production is enough for carcinogenesis.
- Transcriptional silencing does not always require hypermethylation of the entire CpGisland, but methylation of a few specific core CpGdinucleotides may be sufficient.
- Methylation can occur outside the promoter region (long range epigenetic silencing).

## Downregulation of PTEN Primes the Organism for Nutrition-Poor Environment

If PTEN is indeed the 30 gene, how will a downregulated PTEN prime the offspring for a nutrition poor environment? The ideal thrifty gene makes efficient use of available energy to grow and reproduce (64). The information we have about PTEN loss is based on knock out (KO) animal models (65); this leads to complete absence, and may not strictly conform to what occurs when there is more subtle reduction of PTEN expression, as envisioned in this theory.

1. Loss of PTEN is associated with increased self-renewal, cell survival, and proliferation; this is seen in normal human embryonic stem cells (66), as well as cancer stem cells (67).
2. PTEN loss is associated with resistance to starvation, by improved energy efficiency and macropinocytosis (scavenging necrotic cell debris, proteins, and extracellular fluid). Pten mutant Drosophilia flies survived starvation better than Pten wild (68). Similarly, Pten mutant cells proliferate better under conditions of nutrition restriction (69); they switched from hypertrophic growth to hyperplastic growth in one study [though this is point is controversial with some studies showing Pten mutant cells are larger (70)]. Cancer cells deficient in PTEN are resistant to starvation, which is restored by replacing PTEN (71, 72).
3. PTEN deficient cells are more energy efficient. Overexpression of Pten, by introducing an extra Pten, caused these transgenic mice (73, 74) to display increased energy expenditure by activation of brown adipose tissue (BAT) via uncoupling protein 1 (UCP1). Over-expression of PTEN is clearly detrimental in a nutrient-poor environment, resulting in energy wastage. However, it is important to remember this property as insufficient PTEN expression will prevent the organism from “burning off” excess calories, which then has to be stored as fat. (The importance of burning off excess calories is discussed later under the section on “protein leverage.” PTEN inactivation bestowed a bioenergetic advantage to the cells by up-regulating mitochondrial respiratory capacity (75, 76). These cells upregulate oxidative phosphorylation and generate more ATP, wasting less energy in heat production. [However, cancer cells prefers aerobic glycolysis (Warburg effect), presumably to promote flux into biosynthetic pathways (77); despite frequent loss of PTEN].
4. PTEN loss permits safe storage of excess fat. Insulin is primarily a fat storage hormone and higher levels of insulin is a postulated reason for obesity (as per the carbohydrate-insulin model of obesity).“Safe” here means storage without inducing insulin resistance. This is best seen in Cowden syndrome that occurs due to germline mutation of PTEN where insulin sensitivity is maintained despite obesity (78). PTEN loss in sporadic mutations also leads to obesity with retained insulin sensitivity (79), which is consistent with its position downstream of the insulin pathway, inhibiting PI3K/AKT/mTOR pathway. Loss of PTEN has a “brake-off” effect on this pathway, with unrestricted flow of signals at lower serum insulin levels. The major impact of any gene on thriftiness would be modulation of the insulin pathway proportional to availability of nutrition (64), and PTEN fits this role. Women have higher insulin sensitivity despite higher fat mass, apparently due to downregulation of PTEN gene expression in skeletal muscle; however, here the PTEN inactivation is possibly by phosphorylation (80). Incidentally, PTEN deficient preadipocytes derived from lipomas seen in PTEN mutant children have been noted to have increased fat storage (and proliferative) capacity (81); this can be suppressed by inhibitors of mTOR (everolimus) or PI3K (alpelisib) (82). Pten haploinsufficient mice, when given high calorie diet, had lower insulin (and glucose) levels but put on same overall and visceral weight as their wild counterparts (suggesting higher efficiency of the metabolic pathway), with similar increase in cholesterol and triglyceride (83).
5. PTEN deficiency leads to smaller size offspring. As noted by Prentice et al. (2), chronic food shortages end up creating a smaller mother, which imposes a “maternal uterine constraint” on the size of the fetus. Obviously, having a large baby poses serious health risks to the malnourished mother. In mouse models, partial PTEN deletion in the hypothalamus resulted in whole body growth restriction (84, 85). However, this is not universal finding; other studies have reported Pten deficient mice cannot be “physically distinguished” from their litter mates (86).
6. Pten deficiency induces Behavioral changes including hyperphagia (87); it reduces muscle protein degradation (88) and improves endurance (89), both presumably contributing to a survival benefit in a harsh environment. PTEN alteration can also possibly influence Behavior, as loss of PTEN function is implicated in Behavioral disorders such as autism (90).
7. PTEN deficiency in murine oocytes causes the entire oocyte pool to become activated prematurely; this has the effect of females having a maximum of one normal-sized litter before they became infertile at 12–13 weeks of age (91). In nutrition constrained environment, limiting the number of offspring and reducing competition could have its own evolutionary advantages. However, in another study but in adult mice, low protein diet given ad libitum resulted in increased expression of Pten in oocytes and delayed the activation of ovarian primordial follicles (92).
8. Another evolutionary benefit is a shortened lifespan seen in Pten deficient mice, partly attributable to a higher incidence of cancer (93). PTEN can be thought to set the level of “energy entitlement” at conception; the upper limit of what the offspring is eligible to consume through its life in that particular degree of nutrition availability. Consuming more than entitlement is potentially detrimental to other members of the society; in this sense, it can be thought of as a “self-destruct button”.

In summary (Table 1), subtle dose reduction of PTEN proportional to the degree of nutritional deprivation can modify the organism to adapt to a nutrition-poor environment by improving energy efficiency, safe storage of excess energy as fat, and early reproduction. However, this also results in restricted ability to burn off excess calories, resulting in obesity, and its consequences.

**Table 1:** Potential benefits of reduced PTEN expression.

**SR No**	**Benefit**
1	Improved cell proliferation and survival
2	Resistance to starvation
3	Energy efficiency
4	Efficient storage of excess fat
5	Smaller offspring
6	Altered behavior (hyperphagia)
7	Early fertility
8	Reduced life span (quicker turn over)

## Downregulated PTEN Results in Diseases in Nutrient-Rich Environment

The key reason why we are currently concerned with identification of thrifty genes is their postulated role in human disease in a discordant, nutrient-rich environment. The ability of a thrifty gene to reduce energy expenditure and store fat, has distinct survival benefits in a nutrient-poor environment However, excess fat storage can lead to obesity. Fat stored in ectopic locations leads to insulin resistance (IR) and metabolic disorders (type 2 diabetes mellitus, dyslipidemia, cardiovascular diseases) and obesity related cancers (ORCs) in an obesogenic environment. Whether hyperinsulinemia, a marker of IR, is the cause or effect of obesity is still a matter of debate (94).

It has been proposed that the thrifty gene should have the property of plasticity to adapt to a nutrient-rich environment (2) but I believe that it is the inability to adapt that leads to disease.

### PTEN, Obesity and Insulin Resistance

The relationship between obesity, IR and metabolic syndrome is still a matter of discussion and controversy; the two leading hypothesis being the conventional “calorie imbalance (calorie in; calorie out) model” and the more recent “carbohydrate-insulin model.” Whether hyperinsulinemia, a marker of IR, is the cause or effect, is also debated (95). Until this is settled, the role played by PTEN will remain a matter of speculation. It is obvious that PTEN deficiency as proposed in this paper is not compatible with insulin resistance, as PTEN loss is consistently associated with insulin sensitivity [retained even in obese patients of PTEN mutations such as Cowden syndrome (78)].

It is important to understand that fat storage leading to obesity is not *sine qua non* for insulin resistance (IR). It is generally accepted that it is the location, rather than the amount of stored fat that determines IR. Current dogma suggests that IR is due to ectopic fat deposition (96) in locations such as liver [intrahepatic lipids (IHL)], muscles [intramyocellular lipid (IMCL)] or visceral adipose tissue (VAT). Storage in subcutaneous fat (SAT) is considered benign. Liver specific deletion of Pten causes NAFLD (a form of IHL), but these mice remain insulin sensitive and have an overall reduction in body fat (97). This is consistent with the possibility that higher insulin sensitivity in Pten deficient hepatocytes drives glucose into liver cells, increases de no lipogenesis (DNL) and results in fatty liver; presumably peripheral fat is preferentially drawn in and stored in liver.

Similarly, deletion of Pten in adipose tissue (39, 98), skeletal muscle cells (99), pancreatic beta cells (100), or neurons expressing Cre (101) results in insulin sensitivity, despite massive deposits of fat in SAT after high fat diet (HFD) as seen in the last model. Systemic inhibition of Pten in diabetic mice using antisense oligonucleotide resulted in downregulation of Pten in liver (by 90%) and fat (by 75%), reduced insulin levels and restored insulin sensitivity (102).

It is interesting to speculate that PTEN could have a role in body fat distribution and lay the foundations of a metabolically unhealthy organism (such as reduced capacity in subcutaneous adipose tissue forcing storage in the unhealthy visceral adipose tissue); however, the data available is not sufficient to support such a possibility. Deletion of Pten in a subset of adipocytes leads to redistribution of body fat with lipomatosis and partial lipodystrophy (103); however, the changes were not specific to SAT vs. VAT pattern.

Organ of involvement: Does downregulation of PTEN occur in all body tissues or in specific organs (such as liver, muscles or fat)? Downregulation in adipose tissue can explain most of the features of metabolic syndrome. AiPKO mice (39), where Pten was deleted in mature adipocytes, maintained insulin sensitivity despite putting on (mainly)SAT on high fat diet; knocking out Pten after HFD-induced weight gain restored insulin sensitivity and reduced hepatic steatosis.

In a more recent study (104), fat depot specific deletion of Pten induced expansion of local fat depots, with upregulation of Pten in other depots and reduced fat mass there (analogs to deletion of Pten in liver resulting in fatty liver); this is thought to work through an “adipose PTEN—leptin—sympathetic nervous system” activation pathway. The authors suggest that the compensatory upregulation of PTEN in other adipose tissues suggests a “homeostatic set point of PTEN” and the “adipose PTEN-leptin—SNS—PTEN” loop contributes to the maintenance of whole-body adiposity and adipose distribution in adult animals.”

It is tempting to suggest that PTEN downregulation in nutrionally stressed organism occurs preferentially in subcutaneous adipose tissue (SAT), priming the organism for safe fat storage. Evolutionarily, SAT has been the organ around which the “fasting vs. feasting” revolves. As the study by Huang et al. (104) indicates, when this storage capacity is overcome, ectopic fat storage results (96); this is thought that excessive fat storage leads to distension of adipocytes of SAT, activation of adipose triglyceride lipase (ATGL) enzyme, leak of FFA into blood and uptake in other tissues, leading to IR and metabolic syndrome. However, to explain other facets of the thrifty gene hypothesis, such as altered reproduction and increased susceptibility to cancer, deficiency of PTEN in other tissues is essential.

Interestingly, new born Indian babies, though lighter, have increased fat storage (“thin-fat babies”) and this has been correlated with increased risk of metabolic syndrome in adulthood (105). Although the PTEN values in these babies are unknown, the insulin levels are equivalent to a comparable cohort born in London, suggesting greater efficiency of the metabolic pathway (106). (The authors have tried to prove hyperinsulinemia in the Indian babies by adjusting insulin levels to the birth weight, but the serum values (which are more relevant) are not statistically different. The level of clinically relevant glucose value, for example, is “per 100 ml serum,” not “100 ml serum adjusted to body weight”). The authors have suggested that vitamin B12 deficiency and 1-carbon chain abnormalities could result in this thrifty phenotype and has led to the current DM epidemic in India (107). Experimentally, increasing folate levels in breast cancer cell lines lead to increased DNMT1 levels, with promoter methylation of PTEN and its downregulation (and cancer progression) (108). Since pregnant women in India are routinely supplemented with folate (and iron) to prevent neural tube defects, this could be an aggravating factor for the T2DM epidemic in India. In the Pune cohort referred to above, higher maternal erythrocyte folate concentrations at 28 weeks predicted higher offspring adiposity and IR at 6 years of age (109).

Americans have higher rates of T2DM than Europeans (110); Americans also suffer from more side effects of an oral chemotherapy drug called capecitabine. The latter has been linked to higher folate levels in Americans (111) due to food fortification; further investigations are warranted in the relation between folate, PTEN and T2DM.

The relationship between PTEN and insulin sensitivity has been demonstrated in other studies. A Korean study reported that the commonly used oral hypoglycemic agent, metformin, downregulates PTEN via AMPK in preadipocytes and sensitizes them to insulin (112).

Liver specific knock out of Pten in mice lead to fatty liver with maintained insulin sensitivity (100). Obese Zucker Diabetic Fatty (ZDF) rats that display all features of IR and metabolic syndrome (high glucose, insulin, triglycerides and fatty liver) had reduced PTEN expression (mRNA) (by around 40%) in the liver; the same was found in liver of obese humans (38). This study also suggested that unsaturated fatty acids downregulated Pten, which then lead to upregulation of free fatty acid (FFA) transporter CD36 and increased synthesis of triglycerides (setting up a vicious cycle). [Another study by the same team suggested that liver PTEN was downregulated by FFA via micro-RNA-21(113)]. In conclusion, PTEN insufficiency leads to fatty liver (whether by liver specific knockout, where systemic insulin sensitivity is maintained as fat preferentially accumulates in liver; or by obesity *per se*, where excess FFA released by overloaded adipocytes of SAT can downregulate PTEN in liver via FFA; this of course, will be associated with insulin resistance). An organism born with PTEN insufficiency is thus prone to fatty liver, a major component of metabolic syndrome.

It may appear paradoxical but one study showed higher levels (more than 3 fold) of Pten mRNA and protein in skeletal muscle of obese Zucker rats (114), and lean Zucker rats showed upregulation of Pten (2 fold) on high fructose diet in the face of systemic insulin resistance. However, this PTEN upregulation can be interpreted as being protective for the muscle against glucotoxicity and lipotoxicity by dampening down the metabolic pathway. Although there is higher intramyocyte fat content in these obese individuals, there is no equivalent pathological “fatty muscle disease.”

The status of PTEN in humans in relation to obesity and Type 2 diabetes mellitus (T2DM) has not been studied well (115) and the reported data is patchy; for instance:

- A bioinformatics analysis of the search for the CpG islands in the promoter regions of obesity-related genes has identified PTEN as being hypermethylated (116) (downregulation).
- A study from Iran (117) showed that patients with metabolic syndrome were more likely to have methylated PTEN than normal people (downregulation).
- PTEN mRNA is overexpressed in omental tissue (which is considered as VAT) in obese patients with endometrial carcinoma [Table 3, refer Berstein et al. (118)].
- A study on Uyghur Muslims (61) showed lower promoter methylation of PTEN in T2DM patients as compared to normal (3.27 vs. 7.28%), leading to PTEN overexpression.
- A PTEN-polymorphism that results in higher expression of PTEN showed significant correlation with T2DM in Japanese (119), marginal with Chinese Han (120) and none in Danish population (121). This is not an epigenetic change but is included to indicate of how conflicting the data is.

Obviously, the maternal nutrition or intrauterine history of the subjects in the above studies is not known.

Downregulation of PTEN represses the ability of body to burn of excess calories. The ability to burn off excess calories is crucial, because, as per the “protein leverage theory,” organisms will continue to ingest food till a certain level of proteins (especially methionine) is acquired (21). In this obesogenic environment with excess of carbohydrates, getting adequate proteins would mean ingesting large amounts of calories. Inability to burn them would leave the only option of storage, which would be facilitated by an adapted PTEN.

In summary, underexpression of PTEN would promote fat storage due to a more efficient insulin-PI3K/AKT/mTOR pathway, and an inability to burn of excess calories. The reasons for progression to IR must remain speculative. PTEN is certainly not the sole factor determining insulin response. It is possible the PTEN remains underexpressed and is bypassed/ overcome in creation of IR in a nutrient-rich environment. Hyperinsulinemia, which accompanies IR, could be the cause, rather than the effect of obesity (94). The possibility that hyperinsulinemia (present in small for date infants) is needed for growth, and IR is the body's response to protect muscles from glucotoxicity has been discussed by Wells (64). Interestingly, PTEN positive and negative cells proliferate similarly up to a certain level of glucose, but PTEN deficient cells start proliferating rapidly at higher levels (122). Insulin levels rise in response to higher levels of dietary glucose, leading to storage of excess energy as fat (acceptable in adipose tissue) but could lead to a proliferative response in other cells (such as in PTEN deficient); this is detrimental to the organism and IR (by whichever mechanism) could be a protective response by the body to prevent tissues from reacting to high proximate glucose (123). Multiple mechanisms, mostly PTEN-independent, has been postulated as cause of IR; one such is the secretion of Galectin-3 protein by macrophages infiltrating the adipose tissue, which binds directly to insulin receptor and causes IR (124). Fetuin-A, secreted from liver and adipose tissue, can also result impair insulin signaling (125).

It is theoretically possible that the offspring born with low “dose” of PTEN, when faced with a nutrition rich environment, upregulates PTEN, leading to IR and its ill effects. This would imply plasticity of the gene, which is against the basic premise of this hypothesis; obesity related diseases results from a fixed metabolic capacity at birth, and an inability to adapt. PTEN can be upregulated in obesity to create IR via free fatty acids (FFA) (126) and several pro-inflammatory cytokines such as Tumor Necrosis Factor alpha (TNFα) (127) and resisting (128, 129).

The only study aimed at finding PTEN level in relation to maternal diet comes from the laboratory of Dr. Susan Ozanne and has been referred to earlier (25). A maternal low protein diet resulted in increased mammary tumorigenesis in the offspring, with underexpression of PTEN in tumor tissue. A highly palatable diet post weaning induced weight gain but did not increase PTEN in the tumor tissue of the low protein group significantly. Unfortunately, the status of Pten in normal tissue was not reported.

### PTEN and Cancer

The data regarding the role of PTEN in cancer is abundant and reviewed (130, 131) and is consistent; PTEN insufficiency increases cancer susceptibility. This is not surprising as PTEN was initially identified as a Tumor suppressor gene. Reduced “dose” of PTEN increases PIP3 and activates the proliferative PI3K/AKT/mTOR pathway (18). Deficiency of PTEN has been recorded in multiple cancers and Pten knock out animals are good models for tumorigenesis (132). As noted earlier (50), PTEN loss can be seen in adjacent “normal” tissue as well. PTEN expression can be lost due to multiple reasons but methylation leading to repression has been reported in cancers of endometrium (62), gallbladder (52), colon (53), prostate (133), and breast (134).

Studies also show that PTEN deficient animals develop more cancers when exposed to high fat diet, such as of prostate (135) and endometrium (136). Interestingly, most PTEN related cancers are also ORCs (137). Correlation between ORCs and PTEN deficiency in light of the thrifty gene theory suffers from lack of data about *in utero* influences or even weight of the patients. One study on endometrioid endometrial cancer (which is strongly correlated with obesity) showed “increased AMPK phosphorylation and IGFBP2 expression were observed in obese patients with PTEN loss. These findings are markers of nutrient deprivation, which is unexpected in the context of obesity”(138).

PTEN deficiency leads to hyperproliferation and probably plays a permissive role in carcinogenesis; additional events, usually in form of second mutation [e.g., in MYC (139) gene], is needed for malignant transformation—this is possible even in the absence of obesity. In the context of obesity, there are other postulated mechanisms. The role of fibroblast growth factors (FGF) and their receptors (FGFR) is especially compelling. It has been shown that visceral adipose tissue (VAT) produces FGF2 (140) which acts on FGFR to induce transformation of epithelial tissue; interactions involving PTEN in breast (141), endometrial (142), and prostate (143) cancers have been reported but exact nature of these interactions await further clarification.

Interestingly, feeding HFD to female mouse mammary tumor virus-Wnt-1 transgenic (Tg) mice resulted in higher incidence of breast cancers in their offspring; this was associated with downregulation of Pten (144). Similarly, in another mouse study, HFD in dams produced Pten-inactivated prostatic proliferation in the offspring (145). Thus, PTEN deficient cells can turn cancerous when exposed to HFD.

### PTEN and PCOS

Nutrition (lack and excess) has significant effect on reproductive functions (146) and the metabolic syndrome equivalent in the reproductive system is polycystic ovary syndrome (PCOS); these two syndromes tend to overlap (147). It has been suggested that PCOS is likely a result of interactions between genetic predispositions and the modern obesogenic environment (148). Components of this syndrome include obesity, hyperandrogenism, polycystic ovaries, and anovulation (149). The exact cause of this syndrome is still not fully understood.

The role of PTEN in the ovary is pleiotropic and appears to be cell dependent. Essentially, the ovarian follicle has three types of cells—the oocyte, the granulosa cells and the covering theca cells.

a. As seen earlier (91), Pten deletion in primordial follicle lead to premature activation of oocytes and early infertility. However, a chronic low protein diet actually lead to upregulation of Pten in primordial ovarian follicle (via FGF21 and adiponectin) (92) with preservation of fertility; this was in adult mice; the effect of maternal low protein diet on Pten has not been studied (150).
b. In granulosa cells of PCOS patients, PTEN expression was reported as reduced (151) but in another study, insulin was shown to upregulate PTEN in granulosa cells (152). In another study, disruption of Pten in granulosa cells was associated with improved fertility (153).
c. In theca-interstitial cells, Pten deletion produced features of PCOS including hyperandrogenism and early fertility loss (154). Animal models suggest that the PI3K/AKT pathway plays a significant role in the molecular pathology of PCOS (155); the outcome of Pten deletion in theca cells is consistent with this. The involvement of PI3K pathway in PCOS is further demonstrated by the fact that PTEN expression is altered in endometrium of PCOS patients (156).

It would appear that, just in case of cancer, downregulation of PTEN in specific cells of ovary creates a permissive background for PCOS, which is precipitated by a high-nutrition environment. It, of course, remains to be demonstrated that maternal protein restriction results in PTEN downregulation in the ovary, especially the theca cells. Curiously, NAFLD is another condition with PTEN downregulation (in hepatocytes)which is associated with peripheral insulin resistance (38), and both PCOS and NAFLD tend to overlap—in fact, about 40% of the PCOS patients have NAFLD (157). Metformin, which is used with some success in both conditions, is an AMPK activator which induces PTEN (158).

## Differential Expression of PTEN Explains Lack of Consistency in Data

The data regarding PTEN and maternal nutrition, as collated in this paper, is not and cannot be consistent as it would vary depending on experimental conditions. However, it is known that gene expression varies among tissue and it is likely that PTEN expression is not uniformly suppressed across major tissue types (brain, liver, adipose tissue, and muscles) in response to nutritional constraint. Pten haploinsufficient or KO mice are not appropriate models to decide this and unfortunately, there is no low protein (LP) or maternal methionine-choline deficient (MCD) diet models where Pten has been analyzed across different tissues. There is indirect evidence to suggest PTEN expression can be variable. For instance, analysis of metabolically stressed *in vitro* fertilized mice showed acetylation of Txnip gene (involved in metabolism); the adult mice had upregulated mRNA and protein expression of the Txnip as compared to controls (159). But there was differential expression in the tissues—with increased expression of Txnip RNA and protein in adult fat and muscle but not liver or pancreas. TXNIP can activate PTEN by reduction; interestingly, in Txnip KO mice, Pten is inactive in oxidative tissue (skeletal muscle and hearts) but not in lipogenic tissues (liver and adipose tissue) (160). Similarly, in a porcine model (29), low protein diet reduced levels of Dnmt1 and 3b in fetal liver while increasing expression of 3a; but in fetal muscle, Dnmt3a was reduced, Dnmt1 increased with no change in 3b. Target genes were altered and the authors suggest that the “differential DNMT3a and DNMT3b gene expression (in liver and muscle) implicates a tissue-specific impact of maternal diet on both fetal liver and muscle tissue” (29). It would appear that *in utero* epigenetic modifications do not uniformly affect diverse tissues. There could be differential expression depending on various factors such as the availability of redox mechanisms.

## Proving the Hypothesis

There are enough gene expression and methylome profiles existing of both human and animal models of nutrition deprivation that can be analyzed for PTEN expression; the difficulty would be in defining the normal expression since there is no “on” or “off” level, only degrees of activity. It would be particularly interesting to see the Pten levels in normal tissue of the low protein model (25) generated in Dr. Ozanne's laboratory. Experimentally, this can be proven by analyzing PTEN levels in offspring of animals deprived of specific nutrients, especially in organs of interest such as adipose tissue, liver and muscles, and confirming that these changes were brought about by via the methylation arm of the epigenetic pathway. Additional generations can be analyzed to look for transgenerational inheritance.

## Conclusions (Figure 1)

It is proposed that PTEN is the primary thrifty gene (Table 2). Epigenetic modification (methylation) of PTEN promoter suppresses the expression of the gene proportional to availability of nutrients (protein, and possibly choline). This sets the metabolic capacity and adapts the fetus to nutrition availability in post natal environment. A mismatch with calorie abundance results in efficient storage and limited expenditure and causes obesity, metabolic syndrome and cancer. The crux of a thrifty gene is its ability to efficiently use limited energy for growth and reproduction, and store the excess safely, and multiple lines of evidence suggest that PTEN does this brilliantly.

**Figure 1:**
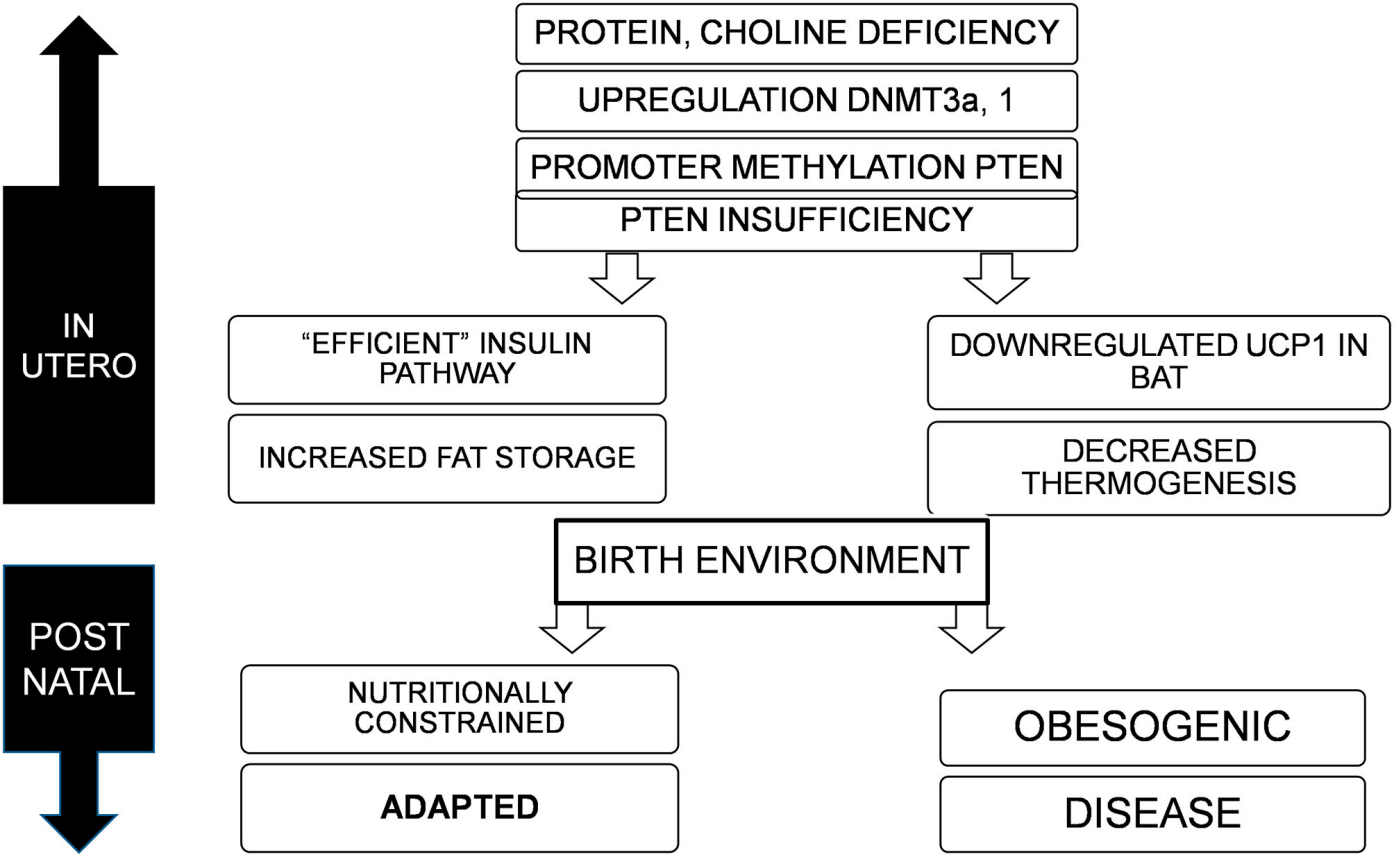
Deficiency of nutrients *in utero*, specifically proteins and choline lead to upregulation of DNMT3a and possibly 1, resulting in promoter methylation and suppression of PTEN, to varying degrees. This adapts the offspring to a nutritionally constrained post natal environment with efficient fat storage and reduced thermogenesis. If the birth environment continues to lack nutrition, the organism is well-adapted for survival, but in an obesogenic environment, would result in obesity, metabolic disorders, and cancer.

**Table 2:** Summary of evidence supporting the hypothesis.

**Postulate**	**Evidence**	**References**
**CAUSE: LOW PTEN DUE TO:**		
1. Specific nutritional deficiency reduces PTEN expression in tissues & offspring	Experimental data with low protein, methionine and choline diets.	(25, 30, 32, 46)
2.This effect is through epigenetic pathway	Experimental data shows upregulation of DNMT3a.	(27, 31)
**EFFECT: LOW PTEN CAN LEAD TO:**		
1. Obesity	Seen in Cowden syndrome.	(78)
	HFD causes obesity with PTEN deleted models.	(39, 101)
	Hypermethylated (downregulated) PTEN is associated with metabolic syndrome.	(116, 117)
2. Fatty liver (NAFLD)	Liver specific deletion causes fatty liver.	(97, 100)
3.Cancer	Tumor suppressor gene.	(130, 131)
	Low expression in multiple obesity related cancers (ORCs).	(52, 53, 62, 133, 134)
4.PCOS	Deletion in theca-interstitial cells leads to features of PCOS.	(154)

## Author Contributions

The author confirms being the sole contributor of this work and has approved it for publication.

## Conflict of Interest

The author declares that the research was conducted in the absence of any commercial or financial relationships that could be construed as a potential conflict of interest.
