# Corrigendum: PTEN: A Thrifty Gene That Causes Disease in Times of Plenty?

**DOI:** 10.3389/fnut.2020.573536

**Published:** 2020-11-17

**Authors:** Ajit Venniyoor

**Affiliations:** Department of Medical Oncology, National Oncology Centre, The Royal Hospital, Muscat, Oman

**Keywords:** PTEN (phosphatase and tensin homolog deleted on chromosome 10), thrifty gene hypothesis, insulin resistance, carcinogenesis, polycystic ovarian disease (PCOD), diabetes mellitus, NAFLD

In the original article, there was a mistake in ^**^Figure 1^**^ as published.

^**^The caption in Box 6 (from top, right branch) reads “INCREASED THERMOGENESIS”^**^.

The corrected ^**^Figure 1^**^ appears below.

**Figure 1:**
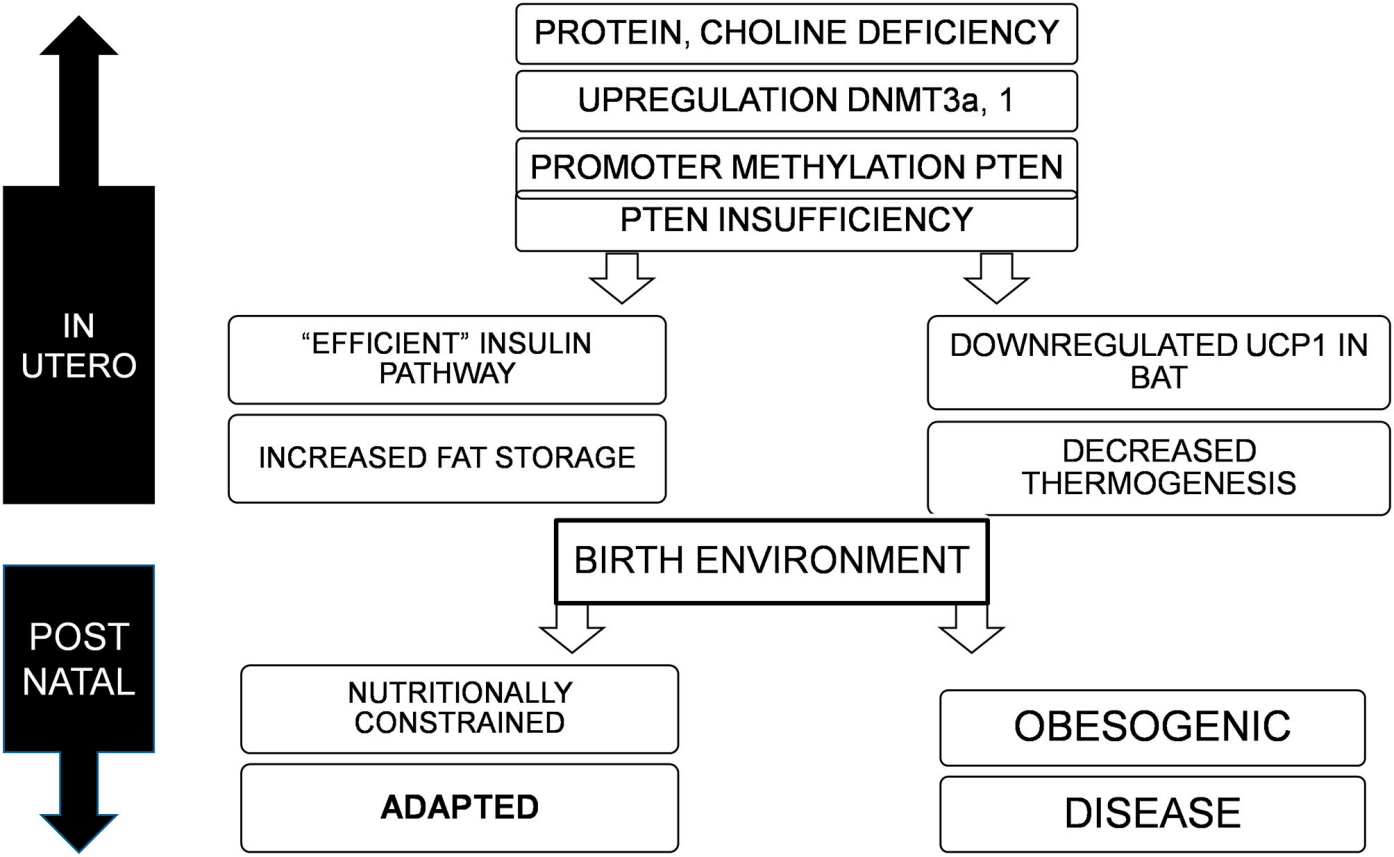
Deficiency of nutrients *in utero*, specifically proteins and choline lead to upregulation of DNMT3a and possibly 1, resulting in promoter methylation and suppression of PTEN, to varying degrees. This adapts the offspring to a nutritionally constrained post natal environment with efficient fat storage and reduced thermogenesis. If the birth environment continues to lack nutrition, the organism is well-adapted for survival, but in an obesogenic environment, would result in obesity, metabolic disorders, and cancer.

The caption should read “DECREASED THERMOGENESIS”.

The authors apologize for this error and state that this does not change the scientific conclusions of the article in any way. The original article has been updated.

